# Regulating Bcl2L12 expression in mast cells inhibits food allergy

**DOI:** 10.7150/thno.34001

**Published:** 2019-07-09

**Authors:** Peng-Yuan Zheng, Xiao-Rui Geng, Jing-Yi Hong, Gui Yang, Jiang-Qi Liu, Li-Hua Mo, Yan Feng, Yuan-Yi Zhang, Tao Liu, Pixin Ran, Zhi-Gang Liu, Ping-Chang Yang

**Affiliations:** 1 Department of Gastroenterology, Fifth Hospital, Zhengzhou University, Zhengzhou, China.; 2 Research Center of Allergy & Immunology, Shenzhen University School of Medicine, Shenzhen, China.; 3 Longgang ENT Hospital & Shenzhen ENT Institute, Shenzhen, China.; 4 Department of Respirology, Third Affiliated Hospital of Shenzhen University, Shenzhen, China.; 5 Department of Pediatric Otolaryngology, Shenzhen Hospital, Southern Medical University, Shenzhen, China.; 6 Department of Otolaryngology, First Hospital and Nursing Collage, Shanxi Medical University, Taiyuan, China.; 7 State Key Laboratory of Respiratory Disease, Guangzhou Medical University, Guangzhou 510006, China.

**Keywords:** Mast cell, inflammation, immunology, apoptosis, interleukin-5.

## Abstract

**Rationale**: Mast cells play a crucial role in allergic diseases. Yet, the regulation of mast cell bioactivities is not fully understood. This study aims to elucidate the role of B cell lymphoma 2 like protein 12 (Bcl2L12), one of the anti-apoptosis proteins, in regulating mast cell apoptosis.

**Methods**: A food allergy (FA) mouse model was developed to establish mast cell over population in the intestinal tissue. Either compound 48/80 (C48/80) or specific antigens were used to activate mast cells in the intestinal mucosa.

**Results**: After treating with C48/80, apoptosis was induced in mast cells of the intestine of naive control mice, but not in FA mice. The expression of Fas ligand (FasL) was lower in the mast cells of FA mice. Interleukin (IL)-5 was responsible for the suppression of FasL by upregulating the expression of Bcl2L12 in mast cells. Bcl2L12 prevented c-Myc, the major transcription factor of FasL, from binding the FasL promoter to inhibit the expression of FasL in mast cells. Inhibition of Bcl2L12 restored the apoptosis machinery of mast cells in the FA mouse intestine.

**Conclusions**: The apoptosis machinery in mast cells is impaired in an allergic environment. Inhibition of Bcl2L12 restores the apoptosis machinery in mast cells in the FA mouse intestine.

## Introduction

The overpopulation of mast cells in local tissue is observed in many inflammatory diseases, such as allergic diseases, chronic inflammation and autoimmune diseases, indicating that mast cells are associated with the pathogenesis of these diseases 1. However, the underlying mechanism of the mast cell over population is not fully understood and needs to be investigated. Mature mast cells contain many granules in the cytoplasm. The components of mast cell granules consist of many chemical mediators, such as histamine, heparin, leukotrienes, serotonin and tryptase. Once released, the chemical mediators induce inflammation in the tissue 2. Mast cells express many receptors on the cell surface, such as Toll-like receptors, immunoglobulin (Ig) receptors and Mas-related genes (Mrgpr; a member of G protein coupled receptor family) 3, 4. Therefore, mast cells are able to recognize various stimuli, or, in other words, mast cells can be activated by multiple factors, such as microbial products, IgE or compound 48/80 3-5, in which those mediated by MrgprB2 can be called a “pseudo allergy” 4. In general, microbe-derived signals induce the chronic activation of mast cells, and chronic inflammation may consequently occur 5, while signals from specific antigens induce anaphylactic mast cell degranulation to evoke allergic reactions 6. Two factors may be attributed to the over population of mast cells in tissues: one is that the mast cell development is strengthened, and another is that the apoptosis machinery of mast cells may be impaired. The abnormal development of mast cells has been extensively investigated 7, 8, yet remains unclear whether the apoptotic machinery in mast cells is impaired in mast cell-related disorders.

Apoptosis is a physiological phenomenon in which body cells undergo programmed death to eliminate senescent cells, damaged cells or cells that are activated but are no longer needed to maintain the homeostasis in the body. Current understandings of apoptosis mechanism are associated with the increases in Fas and Fas ligand (FasL) expression or activation of caspases, in target cells. In certain environments, apoptosis machinery can be impaired. Fox example, the defects of apoptosis were found in peripheral CD4^+^ T cells of asthma patients 9. Interference with the interaction of Fas/FasL can inhibit apoptosis 10. Expression of Fas and FasL was also found in mast cells 11, while whether the expression of Fas/FasL in mast cells under the FA condition is impaired remains to be investigated.

Besides regulation by established programs, apoptosis can also be regulated by many other events, including the intracellular and extracellular factors. The extracellular factors are numerous, such as tumor necrosis factor-α, psychological stress-related events and microbe-derived molecules. The intracellular factors indicate those inside cells and, mainly include the p53 protein and the Fas/FasL system 12. The p53 protein repairs damaged cells and induces apoptosis in those that cannot be repaired. The Fas/FasL pair is one of the systems inducing cell apoptosis 11, while factors regulating mast cell apoptosis in the intestine under FA conditions are not well defined. In this regard, we isolated intestinal mast cells from FA mice and naïve mice. The apoptosis-related gene transcriptome of mast cells was then analyzed. We found that the B cell lymphoma protein 2 like 12 protein (Bcl2L12) was unusually active (Figure. S1 in Supplemental Materials). Published data also indicate that Bcl2L12 plays a critical role in anti-apoptotic machinery development. Early reports indicated that Bcl2L12 played a role in the anti-apoptotic feature of glioma cells, as wells as some other cancers 13. Later studies showed that Bcl2L12 was expressed in other cell types, such as liver cells 14, cardiovascular endothelial cells 15 and immune cells 16 17. Whether Bcl2L12 is associated with the aberrant regulation of mast cell activities (e.g., apoptosis) has not been investigated.

Allergic reactions activate mast cells. Allergic diseases are the result of hyperresponsiveness of the body immune system to innocent antigens, including allergic asthma, allergic rhinitis, allergic dermatitis and food allergy (FA). The immune system produces specific IgE antibodies against specific antigens. IgE binds FcɛRI, the high affinity receptors, on the surface of mast cells to sensitize the mast cell. Re-exposure to specific antigens triggers sensitized mast cells to release chemical mediators to evoke allergic reactions 18. Cumulative reports indicate that the number of mast cells in the tissues with allergic disorders is markedly increased compared to normal tissues 18. Based on the information above, we hypothesize that the apoptosis machinery in the mast cells of subjects with allergic disorders is impaired. To test the hypothesis, an FA mouse model was developed, and the activation-induced mast cell apoptosis in the FA mouse intestinal tissue was characterized.

## Materials and methods

### Mice

Male BALB/c mice (6-8 week old) were purchased from the Guangzhou Experimental Animal Center (Guangzhou, China). The mice were maintained in a specific pathogen-free facility with accessing water and food freely. FasL^-/-^ mice and littermates (both are C57BL/6 background) were purchased from Jackson Laboratory (Bar Harbor, ME). BALB/c background Bcl2L12 knockout (KO) mice were provided by the Animal Institute of Chinese Agricultural Academy; the design procedures of the Bcl2L12 KO mice are presented in the Supplemental Materials. The animal experimental procedures used in the present study were approved by the Animal Ethics Committee at Shenzhen University.

### Development of FA in the mouse intestine

Following our established procedures 19, grouped mice were gavage-fed with ovalbumin (OVA, 1 mg/mouse) mixing with cholera toxin (CT, 20 µg/mouse) weekly for 4 consecutive weeks. To test the allergy status in the intestine, following items were examined:

Serum levels of mMCP-1and OVA-specific IgE. Frequency of mast cells in lamina propria mononuclear cells (LPMC). Levels of Th1 and Th2 cytokines in extracts of intestinal tissue. The core temperature changes upon challenge with specific antigens.

### Immunohistochemistry of intestinal mast cells

Intestinal segments were snap frozen in liquid nitrogen. Frozen sections were prepared and stained with rabbit anti-mouse mMCP-1 (a marker of murine mast cells) and followed by stained with anti-rabbit IgG (labeled with PE) and reagents of FAM-FLICA® Poly Caspase Assay Kit (to stain apoptotic cells). The sections were observed with a fluorescent microscope. The number of mast cells was counted in 20 randomly selected windows, which were averaged as one datum. The sections were coded. The observers were not aware of the code to avoid the observer bias.

### Isolation of mast cells from the intestine

LPMCs were prepared as described above. Mast cells were purified from LPMCs by flow cytometry cell sorting. The FITC-FcεRI (MAR-1) and APC-eFluor-c-kit (2B8) were purchased from eBioscience^TM^ (San Diego, CA) and used as the markers of mast cells. The purity of isolated mast cells was greater than 96% as assessed by flow cytometry.

### Preparation of bone marrow-derived mast cells (BMMC)

Following the established procedures, the bone marrows were collected from BALB/c mouse femurs. The bone marrow cells were prepared and cultured in the presence of IL-3 (30 ng/ml) and stem cell factor (10 ng/ml). The culture medium, including the reagents, was changed in every 3 days. About 2-week culture, BMMCs were purified by flow cytometry cell sorting with the FcεRI and c-kit as the markers of isolation. The purity of isolated BMMCs was greater than 99% as assessed by flow cytometry.

### Assessment of the activation-induced cell apoptosis in intestinal mast cells in mice

Naive mice and FA mice were peritoneal injected (ip) with C48/80 (2.0 mg/Kg in 0.1 ml saline). The mice were sacrificed next day. The small intestine was excised to prepare LPMC; the latter was stained with anti-mMCP1 antibody and FAM-FLICA reagents. The mMCP1^+^ cells were regarded as mast cells. The FAM-FLICA^+^ cells in the gated mMCP1^+^ mast cells were regarded as apoptotic mast cells.

### Overexpression of FasL or c-Myc

The full-length gene sequences of FasL (NM_010177.4) and c-Myc (AH005318.2) were obtained from the Gene Bank. The FasL-expressing (tagged with Flag) or c-Myc-expressing (tagged with His) plasmids were constructed by the Sangon Biotech (Shanghai, China). The plasmids or control plasmids were transfected into BMMCs or HEK293 cells following the manufacturer's instructions. The results of transfection were checked by Western blotting 48 h after.

### Statistics

Data are presented as mean ± SEM (standard error of mean). The difference between two groups was determined by Student *t* test. ANOVA followed by Dunnett's test or Student-Newman-Keuls test was used for multiple comparisons. If necessary, the Pearson correlation assay was performed between two parameters of interest. P<0.05 was considered statistical significance.

Some experimental procedures are presented in supplemental materials.

## Results

### Apoptotic defects are detected in mast cells in FA mouse intestine

**Figure 1:**
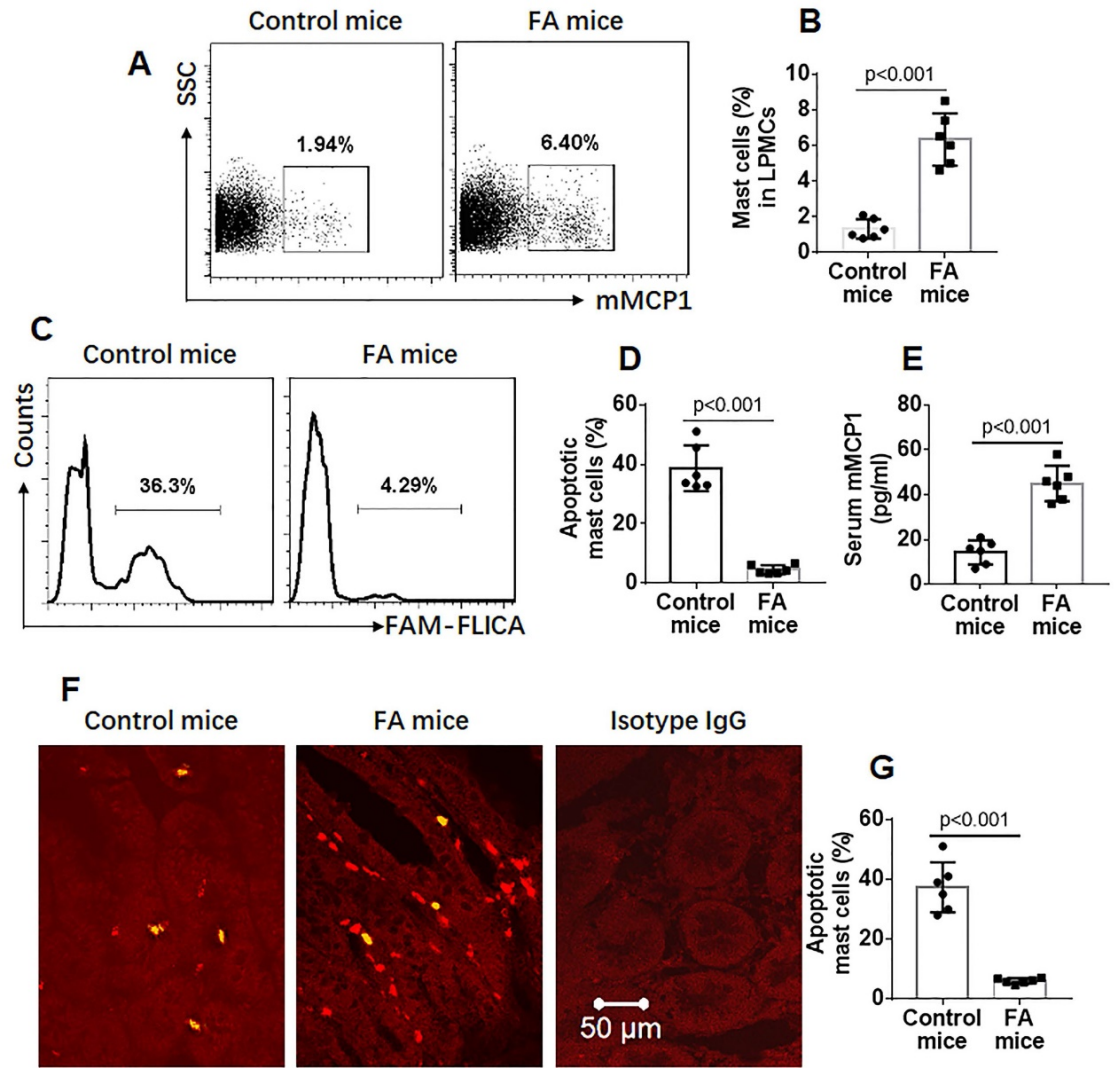
** Mast cells in the intestine of FA mice show apoptosis defects**. FA mice were treated with C48/80 (2.0 mg/kg in 0.1 ml saline) and sacrificed next day. LPMCs were prepared and stained with anti-mMCP1 antibody and FAM-FLICA. The cells were analyzed by flow cytometry. A, gated dot plots indicate frequency of mast cells. B, bars indicate summarized data of the gated dot plots in panel A. C, gated histograms indicate apoptotic mast cells in LPMC. D, bars indicate frequency of apoptotic mast cells in LPMCs. E, bars indicate serum levels of mMCP1. F, representative images show apoptotic (in green) mast cells (in red) in mouse intestine. G, bars show frequency of apoptotic mast cells. Data of bars are presented as mean ± SEM. Each dot inside bars presents data from an independent experiment.

Grouped mice were treated with the OVA/CT procedures 19 to develop FA (Figure S1 in the supplemental materials). To observe the effects of activation on inducing mast cell apoptosis, both FA and control groups were treated with a non-specific mast cell activator, C48/80 [mouse intestinal mast cells express MrgprB2 4 (Figure S3), the receptor of C48/80 [4, 20]] to induce mast cell activation. The mice were sacrificed the next day. Lamina propria mononuclear cells (LPMCs) were prepared and stained with anti-mMCP1 antibody and the FAM-FLICA® Poly Caspase Assay reagents. The cells were analyzed with a flow cytometer. About 1.94% mast cells were detected in LPMCs of control mice while about 6.4% mast cells were detected in LPMCs of FA mice (Figure 1A-B). Further analysis showed that about 38.7% apoptotic mast cells were detected in naïve control mice while only 4.6% apoptotic mast cells were found in FA mice (Figure 1C-D), which were in parallel to serum mMCP-1 levels (Figure 1E). The data were verified by immunohistochemistry analysis, and about 37.3% apoptotic mast cells in naïve control mice and 6% apoptotic mast cells in FA mice were observed (Figure 1F-G). The results indicate that mast cells in the FA mouse intestine have apoptosis defects. Besides activating mast cells, C48/80 also induces mast cell apoptosis. To verify the results, we generated bone marrow-derived mast cells (BMMCs; Figure S4). BMMCs were exposed to C48/80 in culture for 24 h. Indeed, exposure to C48/80 *in vitro* also induced BMMC apoptosis in a dose-dependent manner (Figure S5).

### Mast cells in FA mouse intestine express lower levels of FasL after activation by C48/80

**Figure 2:**
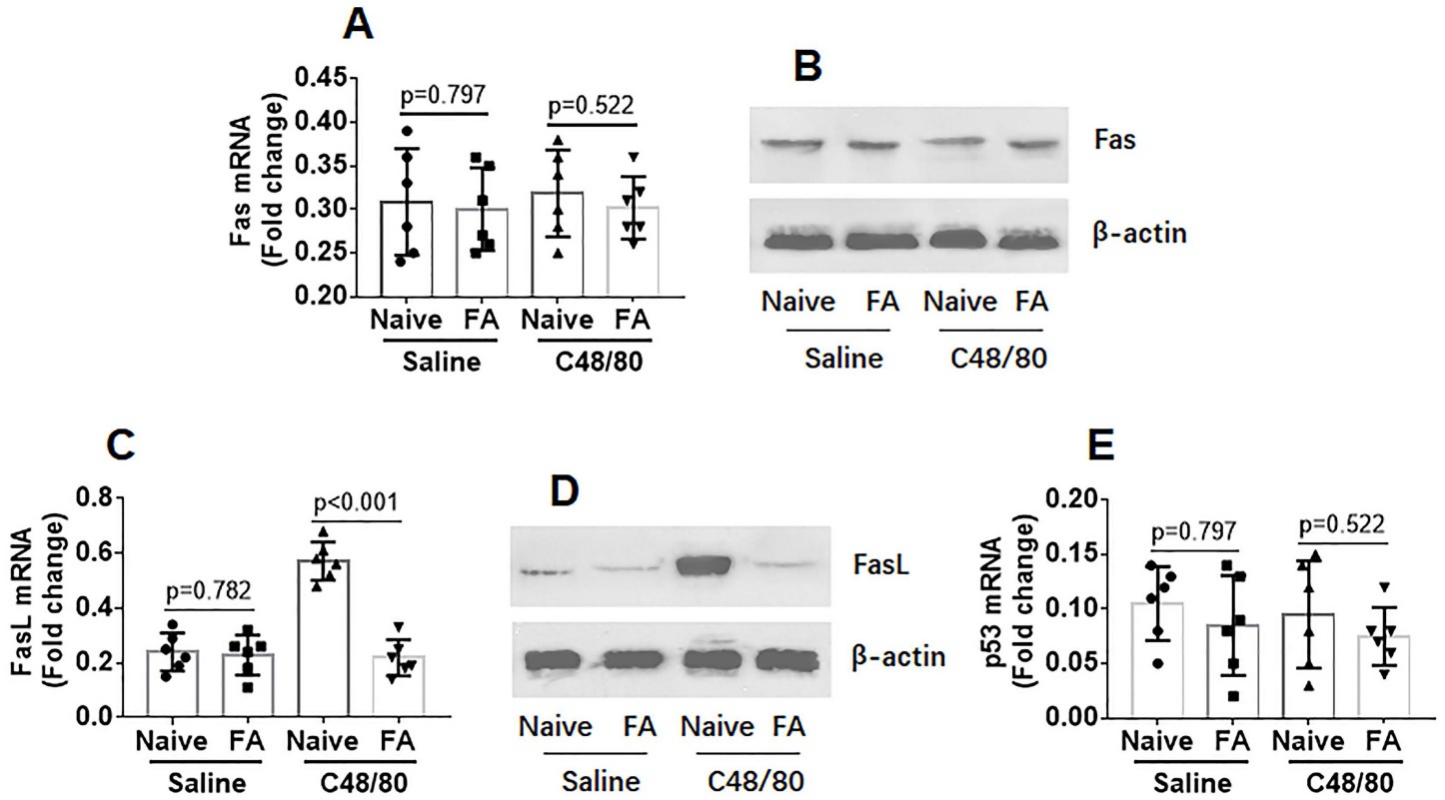
** FA suppresses activation-induced FasL expression in mast cells**. Naïve mice and FA mice were treated with saline or C48/80 (ip) and sacrificed next day. Mast cells were isolated from LPMCs by MACS and analyzed by RT-qPCR and Western blotting. A-B, levels of Fas mRNA (A) and Fas protein (B) in mast cells. C-D, levels of FasL mRNA (C) and FasL protein (D) in mast cells. E, levels of p53 mRNA in mast cells. Data of bars are presented as mean ± SEM. Each dot inside bars presents data from an independent experiment.

**Figure 3:**
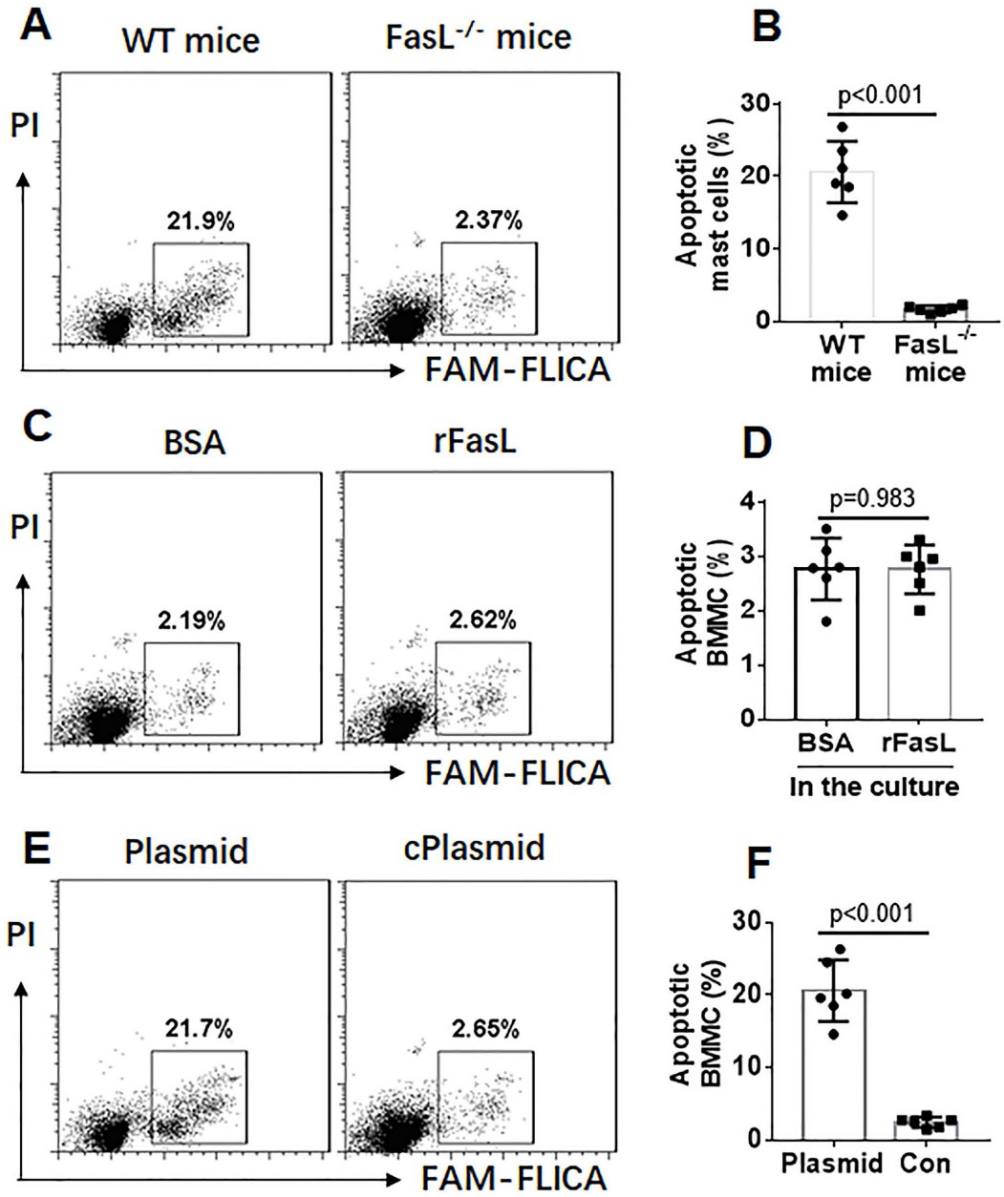
** FasL is required in the C48/80-induced mast cell apoptosis**. A-B, wild type (WT) mice (n=6) and FasL^-/-^ mice (n=6) were treated with C48/80 as described in the text. Intestinal mast cells were isolated by MACS. C-D, BMMCs were treated with rFasL (1 µg/ml) or BSA (1 µg/ml) for 24 h. E-F, BMMCs were transfected with FasL-expressing plasmids or control plasmids (cPlasmid). The cells were analyzed by flow cytometry. Gated dot plots indicate frequency of apoptotic mast cells. Bars indicate summarized data of apoptotic mast cells. Data of bars are presented as mean ± SEM. Each dot represent data from one mouse.

The data of Figure 1 suggest that the apoptosis machinery in mast cells of FA mice is impaired after activating by C48/80. Since Fas and FasL are the signature molecules in activation-induced apoptosis 21, we assessed the expression of Fas and FasL in mast cells isolated from LPMCs. As shown by data of RT-qPCR and Western blotting, the expression of Fas in intestinal mast cells was not significantly different between control mice and FA mice (Figure 2A-B). However, expression of FasL was markedly increased in mast cells of the control group, which was much less in FA mice after exposure to C48/80 *in vivo* (Figure 2-CD). Because p53 is also involved in mast cell apoptosis 22, we assessed the expression of p53 in mast cells. The results showed that exposure to C48/80 did not significantly alter the expression of p53 in mast cells (Figure 2E). The results suggest that the suppression of the FasL expression may be associated with the apoptosis defects of mast cells in FA mice. To verify the inference, FasL^-/-^ mice and wild type (WT) mice were treated with C48/80 (ip). C48/80 administration did not induce intestinal mast cell apoptosis in FasL^-/-^ mice (Figure 3A-B). Since FasL can be produced by other cell types, such as T cells 23, whether exogenous FasL can induce mast cell apoptosis is unclear. To verify this, BMMCs were prepared and exposed to recombinant FasL in culture for 48 h. However, no apparent apoptosis was induced in the BMMCs (Figure 3C-D). This suggests that the C48/80-induced apoptosis of mast cells is triggered by endogenous FasL. To verify the inference, a FasL-expressing plasmid was constructed by Sangon Biotech (Shanghai, China) and transfected into BMMCs to make BMMCs overexpress FasL (Figure S6). As shown by flow cytometry, endogenous FasL markedly induced apoptosis in BMMCs (Figure 3E-F). Taken together, the results demonstrate that exposure to C48/80 can activate mast cells and induce mast cell apoptosis, in which the over expression of FasL plays a critical role.

### IL-5 is negatively associated with the expression of FasL in mast cells

To investigate the role of Th2 polarization in inducing apoptosis defects in mast cells, we collected intestinal samples from FA and control mice. Protein extracts were prepared. Th2 cytokine levels in the extracts were determine by ELISA. The levels of IL-4, IL-5 and IL-13 were higher in FA mice than those in naïve control mice (Figure S2E-G), among which the IL-5, but not IL-4 or IL-13, levels were negatively correlated with FasL expression in mast cells (Figure S7). The results suggest that IL-5 may be associated with the lower expression of FasL in mast cells of FA mouse intestine.

### IL-5 suppresses FasL expression in mast cells

**Figure 4:**
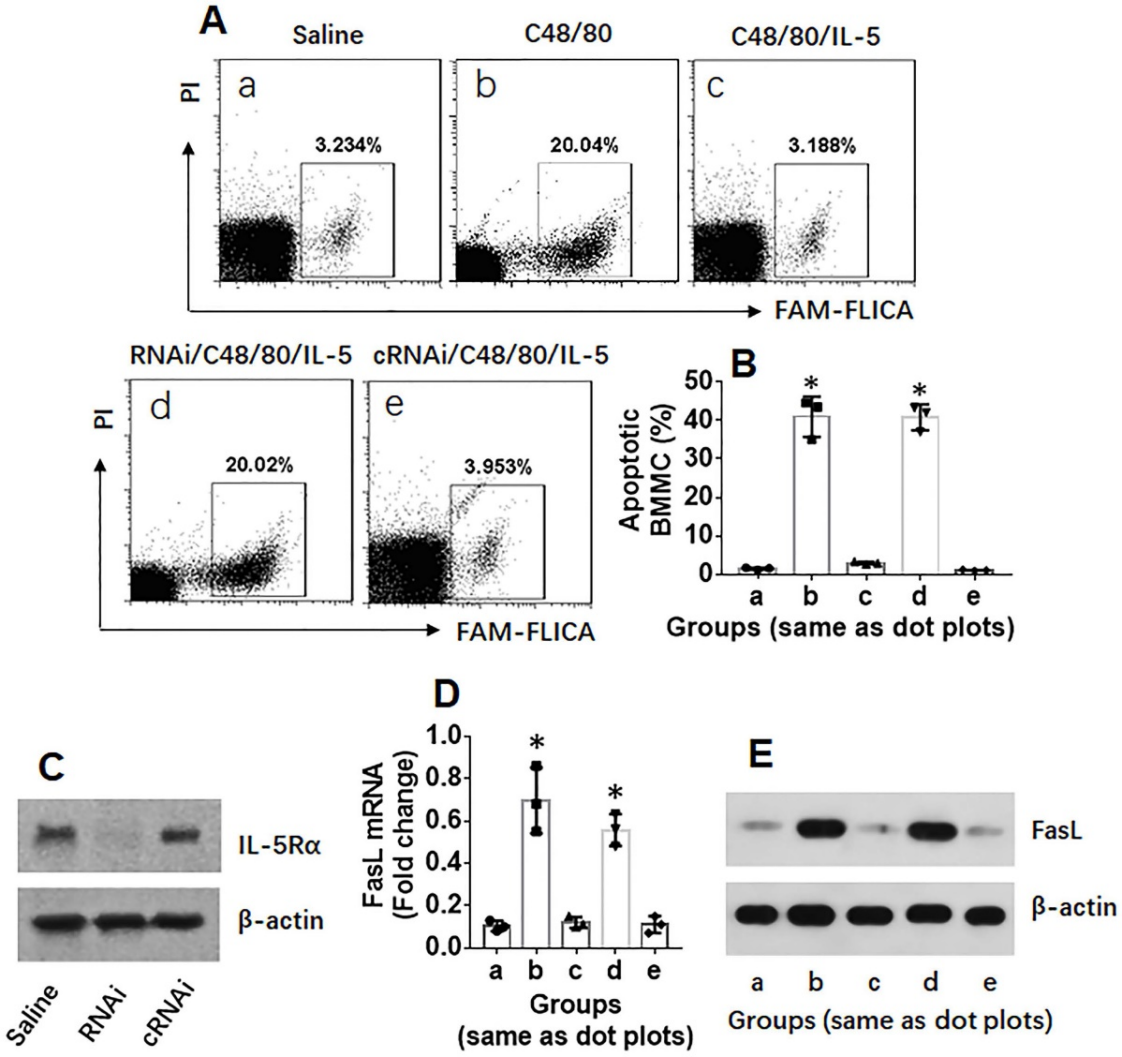
** IL-5 suppresses FasL expression in mast cells**. BMMCs were treated with procedures denoted above each dot plot panel. C48/80: 4 µg/ml in the culture. IL-5: 1 µg/ml. RNAi (cRNAi): BMMCs were treated with IL-5 receptor-α (IL-5Rα) RNAi (or control RNAi). A, gated dot plots show frequency of apoptotic BMMCs. Bars show summarized apoptotic cells. B, bars indicate summarized data of apoptotic BMMCs in panel A. C, results of IL-5α RNAi. D, bars indicate FasL mRNA levels in BMMCs. E, immunoblots indicate FasL protein in BMMCs. Data of bars are presented as mean ± SEM. Each dot represent data from one experiment. *, p<0.001, compared with group a.

To investigate the mechanism by which IL-5 suppresses FasL expression in mast cells, BMMCs were stimulated with C48/80 in culture for 24 h, and the cells were analyzed by flow cytometry, RT-qPCR and Western blotting. The results showed that exposure to C48/80 induced BMMC apoptosis as well as increase the expression of FasL, which did not occur in FasL-deficient BMMCs. To observe the role of IL-5 in fasL suppression in mast cells, BMMCs were exposed to both IL-5 and C48/80 in culture for 24 h. The C48/80-induced increase in FasL expression and apoptosis in BMMCs was blocked by IL-5; such an effect was abolished by knocking down the IL-5Rα (Figure 4). The results demonstrate that IL-5 can suppress FasL expression in BMMCs.

### Bcl2L12 mediates effects of IL-5 on FasL suppression in mast cells

**Figure 5:**
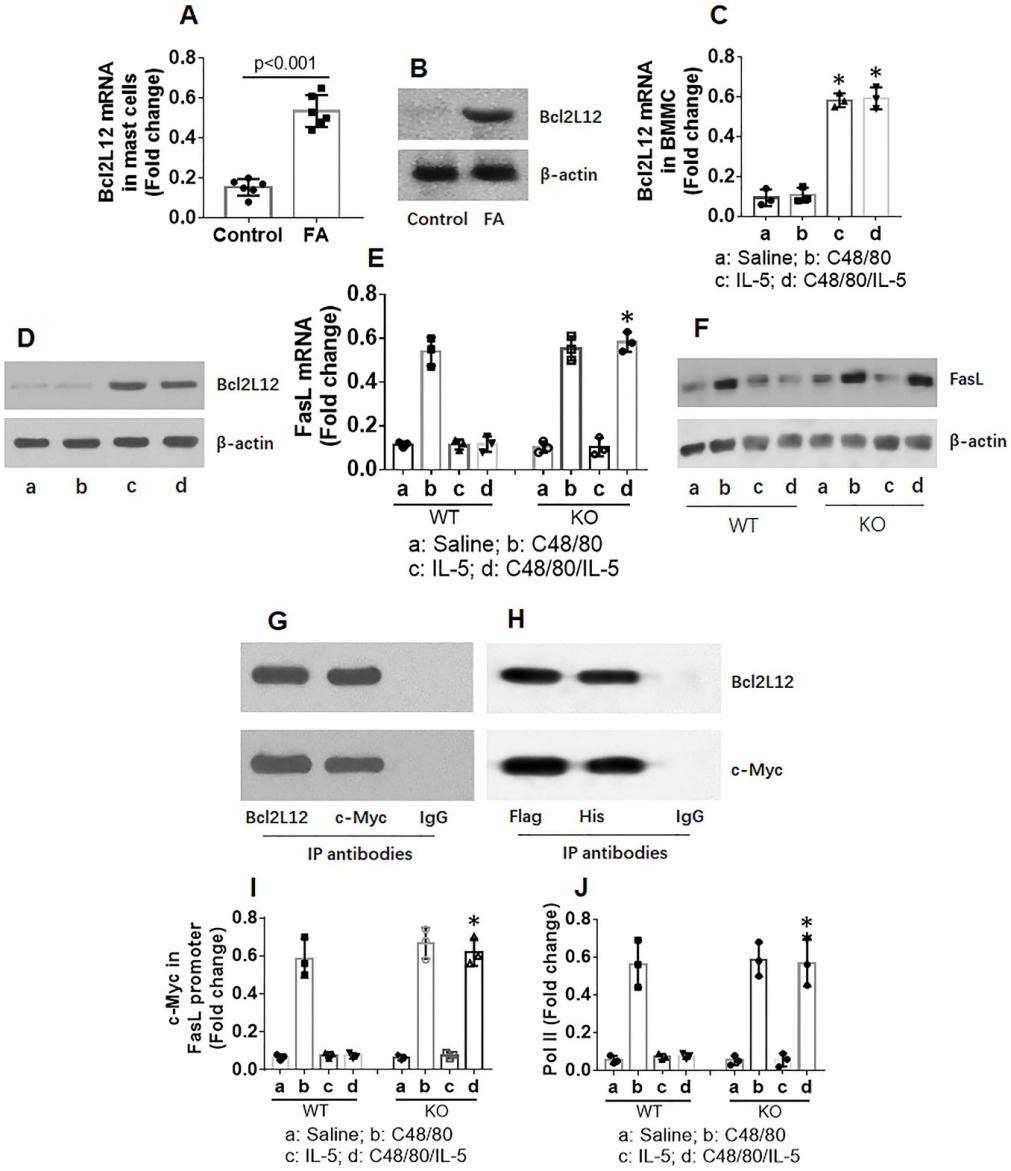
** Bcl2L12 suppresses FasL expression in mast cells**. A-B, mast cells were isolated from mouse intestine and analyzed by RT-qPCR and Western blotting. Bars indicate Bcl2L12 mRNA expression levels. Immunoblots indicate Bcl2L12 levels. C-G, BMMCs were treated with the procedures denoted below the histograms of panel C. C-D, expression of Bcl2L12 in BMMCs. E-F, expression of FasL in BMMCs. G, a complex of Bcl2L12 and c-Myc in mast cells isolated from FA mouse intestine (by co-IP). H, a complex of recombinant Bcl2L12 and recombinant c-Myc in HEK293 cells (by co-IP). I-J, c-Myc and pol II levels at the FasL promoter locus. Data of bars are presented as mean ± SEM. Each dot represent data from one mouse or one experiment. *p<0.001, compared with the saline group (C) or WT group (E, I and J).

Because Bcl2L12 plays a role in inducing an anti-apoptotic feature in cancer cells 13, we sought to determine whether Bcl2L12 was also involved in inducing apoptotic defects in mast cells. The data showed that Bcl2L12 levels were increased markedly in mast cells of FA mouse intestine (Figure 5A-B) and in BMMCs after exposure to IL-5 (Figure 5C-D). Exposure to C48/80 did not increase Bcl2L12 expression (Figure 5C-D). To observe whether Bcl2L12 is associated with FasL suppression in mast cells, we stimulated Bcl2L12 KO BMMCs with C48/80 or/and IL-5 in culture. Knockout of Bcl2L12 blocked the IL-5-induced FasL suppression in BMMCs (Figure 5E-F). Bcl2L12 formed a complex with c-Myc, the major gene transcription factor of FasL 24, 25, in BMMCs after exposure to IL-5/C48/80 (Figure 5G). The results were verified since recombinant Bcl2L12 and recombinant c-Myc formed a complex in HEK293 cells (Figure 5H). The physical contact of Bcl2L12 and c-Myc prevented c-Myc from binding the FasL promoter (Figure 5I) as well as suppressed the levels of RNA polymerase II (Pol II; Figure 5J), an indicator of gene transcription, in BMMCs. The results demonstrate that IL-5 increases expression of Bcl2L12 in mast cells; Bcl2L12 restricts expression of FasL in mast cells.

### Inhibition of Bcl2L12 restores apoptosis machinery in mast cells in the mouse intestinal mucosa

**Figure 6:**
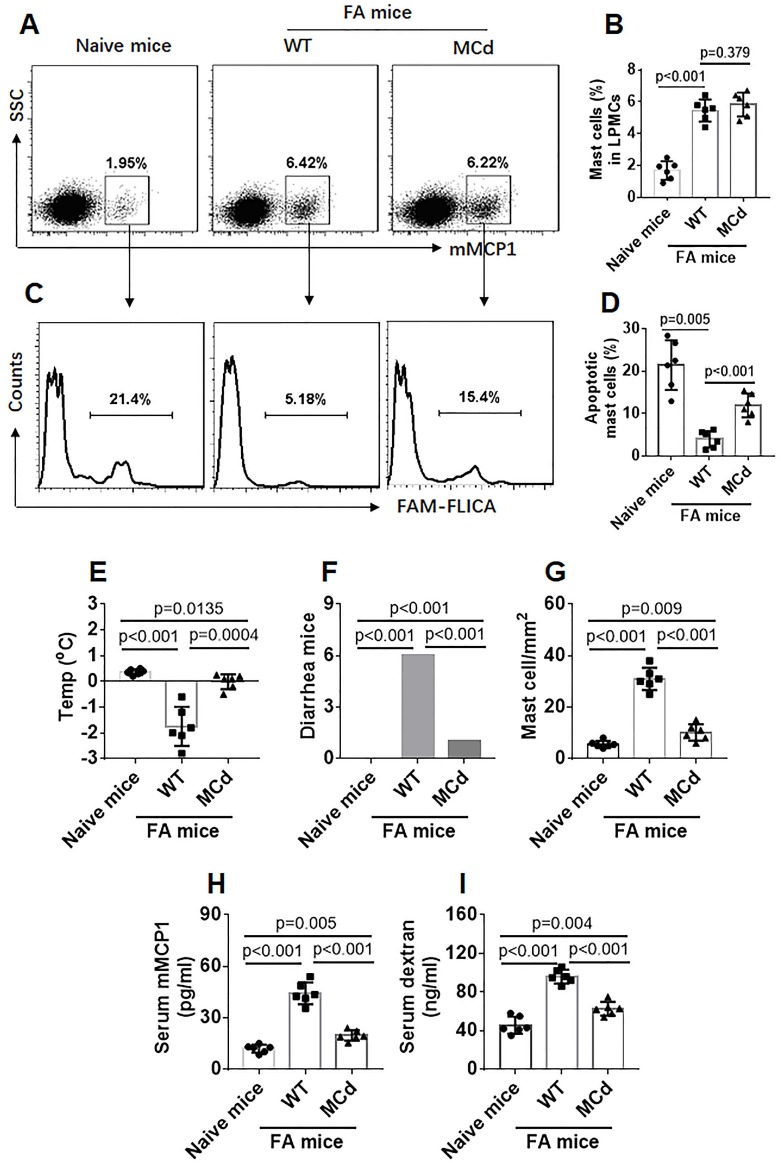
** Inhibition of Bcl2L12 restores apoptosis machinery in mast cells of FA mice**. Wild type (WT) mice and mice with mast cell-deficient of Bcl2L12 (MCd) were sensitized to OVA. A-D, mice were treated with C48/80 (ip) and sacrificed next day. LPMCs were prepared and analyzed by flow cytometry. A, gated dots indicate frequency of mast cells in LPMCs. B, bars indicate summarized mast cell frequency in LPMCs. C, gated histograms indicate apoptotic mast cells in LPMCs. D, bars indicate summarized apoptotic mast cells in LPMCs. E-H, sensitized mice were treated with C48/80 (ip) two times with 48 h apart; the mice were sacrificed 24 h after the second time treating with C48/80. E-F, bars indicate core temperature changes (E, recorded 30 min after the injection with C48/80) and number of mice had diarrhea (F, recorded 0-2 h after the injection with C48/80). G, bars indicate mast cell counts in the intestinal mucosa (representative images are presented in Fig S6). H, bars indicate serum levels of mMCP1. I, sixteen hours after C48/80 ip, mice were gavage-fed with TRITC-dextran and sacrificed 4 h after. Blood samples were collected. Bars indicate serum levels of TRITC signal. Data of bars are presented as mean ± SEM. Each dot inside bars presents data from an independent experiment.

Data in Figure 5 indicate that Bcl2L12 induces apoptosis defects in mast cells, suggesting that inhibiting Bcl2L12 may restore the apoptosis machinery in mast cells of FA subjects. To test this, an FA mouse model was developed with wild type (WT) mice and mice with Bcl2L12-deficient mast cells (MCd). After sensitization, the mice were treated with C48/80 to activate the mast cells. LPMCs were isolated from mice and analyzed by flow cytometry. Although the total number of mast cells was comparable between the WT mice and MCd mice (Figure 6A-B), the frequency of apoptotic mast cells was significantly higher in MCd FA mice than that in WT FA mice (Figure 6C-D). The mice were re-treated with C48/80 two days later. Mast cells were counted in the intestinal tissue by immunohistochemistry. The results showed that the frequency of mast cells was much less in MCd mice than that in WT mice (Figure 6E; Figure S8). The FA response (change of core temperature, diarrhea, serum mMCP1 levels and intestinal epithelial barrier function) was much less in MCd mice than that in WT mice. The results demonstrate that inhibition of Bcl2L12 can restore the apoptotic machinery in mast cells of FA mice, which efficiently attenuates FA response.

## Discussion

We observed that apoptosis could be induced in mast cells after activation in physiological conditions, but not in an allergic environment. IL-5, one of the Th2 cytokines, increased the expression of Bcl2L12 in mast cells. Bcl2L12 formed a complex with c-Myc, the transcription factor of FasL, in mast cells to restrict the expression of FasL. Inhibition of Bcl2L12 restored the apoptosis machinery in mast cells of FA mice and attenuated mast cell-related FA response.

Mast cells play a critical role in the pathogenesis of allergic inflammation and other types of chronic inflammation, such as inflammatory bowel disease 26. Mast cells also have physiological functions—that is, they help to cure of wounds, expel pathogens, etc. Mast cells express Toll like receptors (TLR) 27 that can recognize the stimuli of pathogens, including parasites, bacteria and viruses. In other words, mast cells can be activated by exposure to pathogens. Once activated, mast cells release many chemical mediators to expel the invaded pathogens 28. The present data show that apoptosis occurs in mast cells after activation by C48/80. This phenomenon demonstrates that apoptosis can be triggered in mast cells after activation to terminate the release of chemical mediators to avoid inducing tissue injury and inflammation. The present data show that after activation, mast cells from a naïve mouse intestine underwent apoptosis, while the FA mouse intestine had less apoptotic mast cells, suggesting there is a link between the over population of mast cells in the intestinal mucosa of FA mice and the apoptosis defects in mast cells.

The data show that exposure to IL-5, one of the Th2 cytokines, prevents the induction of apoptosis in mast cells by inducing the expression of Bcl2L12. Over production of Th2 cytokines is one of the features of allergic diseases and some other inflammations 29. IL-5 is the key cytokine to promote the development of eosinophils 30. The present data add novel information to the characteristics of IL-5; IL-5 not only regulates eosinophil development, but it also regulates mast cell activities by inducing the expression of Bcl2L12 in mast cells.

Previous studies show that mast cells express FasL 11. Our data are in line with the data; we also found that intestinal mast cells and BMMCs expressed FasL, which could be up regulated by activation. In this study, exposure to C48/80, the non-specific activator, induced expression of FasL in mast cells. The Fas/FasL system plays a central role in the apoptosis machinery 11. Inhibition of Fas/FasL impairs the apoptotic machinery 31. The present study has revealed a novel factor, Bcl2L12, in the suppression of apoptosis in mast cells through regulating the expression of FasL at gene transcription levels.

Bcl2L12 is a member of the anti-apoptotic family. By suppressing p53 protein, caspase 3 and caspase 7, Bcl2L12 exerts its role of anti-apoptosis. Early studies found that Bcl2L12 was mainly involved in the pathogenesis of cancers 13. Later, it was found that Bcl2L12 was also associated with activities of other cell types 14, 15. Our previous studies found that Bcl2L12 was involved in immune deregulation by interfering with the expression of IL-10 in peripheral B cells of patients with allergic rhinitis 16 or inflammatory bowel disease 32 and facilitating the development of Th2 polarization 17. The present data add novel information to the pathogenesis of disorders with mast cells—that is, IL-5 and Bcl2L12 play a role in the development and maintenance of mast cell over population in the local tissue. It is noteworthy that Bcl2L12 KO mice still have mast cells in the intestine in similar amounts compared with WT mice, indicating that Bcl2L12 is not involved in the lineage development of mast cells.

Mast cells are the main effector pro-inflammatory cells in allergic diseases. By releasing chemical mediators, mast cells induce an allergic response and inflammation in the local tissue or induce systemic response such as anaphylaxis 1. The present data show that normal tissue also has mast cells. These mast cells can be activated by proper stimulus. Since mast cells can be activated by multiple factors 5 and can be activated under physiological conditions, there should be proper remedies to terminate mast cell activation to avoid inducing tissue injury. Our data provide mechanistic evidence that activation can induce mast cell apoptosis, indicating that mast cells can be eliminated soon after activation.

The data show that fewer mast cells in FA mouse intestine can be induced apoptosis. The phenomenon suggests that mast cells in the FA mouse intestine have apoptosis defects. In other words, mast cells in the FA mouse intestinal mucosa have longer life spans than those in normal mice. This may be one of the reasons for the mast cell overpopulation found in FA mouse intestinal mucosa. The present data show the causative factor for this phenomenon; mast cells of the FA mouse intestinal mucosa over express Bcl2L12, and Bcl2L12 is an anti-apoptotic protein that confers mast cells with its anti-apoptotic ability.

Stabilizing the mast cell membrane can prevent mast cell activation. Administration of mast cell stabilizers can efficiently inhibit allergic response to prevent allergy attacks 33. This information mirrors the importance of mast cells in the pathogenesis of allergic diseases. By revealing the role of Bcl2L12 in the induction of apoptotic defects in mast cells, the present study suggests that inhibition of Bcl2L12 restores the apoptosis machinery in mast cells of FA mice. The inference is supported by further experimental data that activation by C48/80 efficiently induces mast cell apoptosis in MCd FA mice, but not in WT FA mice. Importantly, the second activation by administration of C48/80 did not induce mast cell activation-related FA response in MCd mice in contrast to WT mice, suggesting the therapeutic potential in the treatment of allergic diseases by inhibition of Bcl2L12 in mast cells.

The limitation of this study is that we knocked out the Bcl2L12 gene from mast cells used in c-Kit-Cre mice to delete the loxP-sandwiched gene segment. In fact, c-Kit is not solely expressed by mast cells, but also expressed by other cells, such as hemopoietic progenitors, melanocytes, spermatogonia, oocytes, and some natural killer cells. Thus, the expression of Bcl2L12 is also affected in those cells. Fortunately, the lack of Bcl2L12 expression in those cells has less influence on mast cell apoptosis machinery.

In summary, the present study shows that apoptosis can be induced in mast cells by activation under physiological condition. Over production of IL-5 increases the expression of Bcl2L12, and the latter interferes with the expression of FasL and compromises the apoptosis machinery in mast cells. Inhibition of Bcl2L12 restores apoptosis machinery in intestinal mast cells in FA mice, suggesting that inhibiting Bcl2L12 in mast cells has potential clinical application in the treatment of allergic diseases.

## Supplementary Material

Supplementary methods and figures.

## Figures and Tables

**Table 1:** Primers used in the present study

Molecule	Forward	Reverse
Bcl2L12	ttccgagttctatgccctgg	ccagtttacgatgcagagcc
Fas	acctccagtcgtgaaaccat	ctcagctgtgtcttggatgc
FasL	atagccaaccccagtacacc	gctggttgttgcaagactga
c-kit	cccgacgcaacttccttatg	agctcaggaccttcagttcc
FcɛRI	agaggccacactgaatgaca	cgtgtccacagcaaacagaa
MrgprB2	cctcagcctggaaaacgaac	cccaggaaccacagcactat
β-actin	ggaaatcgtgcgtgacatca	gccacaggattccataccca
