# Supplementary material for: Regulating Bcl2L12 expression in mast cells inhibits food allergy
Source: Theranostics. 2019 Jul 9;9(17):4982–92. doi: 10.7150/thno.34001 (PMC6691383; doi:10.7150/thno.34001)
Supplement: Supplementary file 1 — Supplementary methods and figures. [file thnov09p4982s1.pdf]

Supplemental Materials

To regulate Bcl2L12 expression in mast cells inhibits food allergy

Running title: Bcl2L12 prevents mast cell apoptosis

Peng-Yuan Zheng[1]*, Xiao-Rui Geng[2,3]*, Jing-Yi Hong*[2,4], Gui Yang[2,3], Jiang-Qi Liu[2,3], Li-Hua Mo[5], Yan Feng[6], Yuan-Yi Zhang[2], Tao Liu[6], Pixin Ran[7], Zhi-Gang Liu[2], Ping-Chang Yang[2]

1, Department of Gastroenterology, Fifth Hospital, Zhengzhou University, Zhengzhou, China. 2, Research Center of Allergy & Immunology, Shenzhen University School of Medicine, Shenzhen, China. 3, Longgang ENT Hospital & Shenzhen ENT Institute, Shenzhen, China. 4, Department of Respirology, Third Affiliated Hospital of Shenzhen University, Shenzhen, China. 5, Department of Pediatric Otolaryngology, Shenzhen Hospital, Southern Medical University, Shenzhen, China. 6, Department of Otolaryngology, First Hospital and Nursing Collage, Shanxi Medical University, Taiyuan, China. 7, State Key Laboratory of Respiratory Disease, Guangzhou Medical University, Guangzhou 510006, China.

*Equally contributed to this work

Corresponding authors. Dr. Ping-Chang Yang (pcy2356@szu.edu.cn) and Dr. Zhi-Gang Liu (lzg@szu.edu.cn). Room A7-509 in Xili Campus, Shenzhen University School of Medicine, 1066 Xueyuan Blvd, Shenzhen 518055, China. Tel: 75581672722. Fax: 75586671906.

Reagents

Antibodies of Bcl2L12 were purchased from abcam (Cambridge, MA). ELISA kits of IL-4, IL-5, IL-13, IFN-γ, total IgE, OVA-specific IgE and mMCP-1 were purchased from Biomart (Beijing, China). Recombinant IL-5 protein was purchased from R&D Systems (Minneapolis, MN). Recombinant FasL protein was purchased from XMJ Scientific (Beijing, China). Antibodies of mMCP1, Fas, FasL, c-Myc, c-kit, FcεRI and IL-5Rα RNAi kit were purchased from Santa Cruz Biotech (Santa Cruz, CA). The customized anti-MrgprB2 poly clonal antibody (labeled with or without FITC) was produced by Sangon Biotech (Shanghai, China). Reagents and materials for RT-qPCR and Western blotting were purchased from Invitrogen (Carlsbad, CA). Compound 48/80, reagents for immunoprecipitation (IP) and chromatin IP (ChIP) were purchased from Sigma Aldrich (St. Louis., MO). FAM-FLICA® Poly Caspase Assay Kit was purchased from ImmunoChemistry Technologies, LLC (Bloomington, MN). Immune cell isolation reagents were purchased from Miltenyi Biotech (San Diego, CA).

Isolation of LPMC from intestine

Intestinal segments were excised immediately after the sacrifice. The tissues were cut into small pieces (2 × 2 × 2 mm) and incubated with collagenase IV (1 mg/ml) at 37 °C for 2 h with mild agitation. Single cells were passed through a cell strainer (70 µm first, then 40 µm). The mononuclear cells were isolated by the Percoll gradient density centrifugation and cultured in RPMI1640 medium. The viability of the isolated cells was greater than 98% as assessed by Trypan blue exclusion assay.

Cell culture

Immune cells were cultured in RPMI1640 medium supplemented with 100 U/ml penicillin, 0.1 mg/ml streptomycin, 2 mM glutamine and 10% fetal bovine serum. The medium was changed in 1-2 days. The viability of the cells was greater than 99% as determined by Trypan blue exclusion assay.

Enzyme-linked immunosorbent assay (ELISA)

Cytokine levels in the serum and culture supernatant were determined by ELISA with commercial reagent kits following the manufacturer's instructions.

Real-time quantitative RT-PCR (RT-qPCR)

Cells were obtained from relevant experiments. The total RNA was extracted from the cells with the TRIzol reagents. The cDNA was synthesized from the RNA with a reverse transcription kit following the manufacturer's instructions. Samples were amplified in a qPCR device with the SYBR Green Master Mix and the presence of relevant primers as presented in Table 1. The results were presented as fold change against the housekeeping gene β-actin.

Western blotting

The total proteins were extracted from cells obtained from relevant experiments, fractioned by SDS-PAGE (sodium dodecyl sulfate–polyacrylamide gel electrophoresis),

and transferred onto a PVDF membrane. After blocking with skim milk solution (5%) for 30 min, the membrane was incubated with the primary antibodies of interest overnight at 4 °C, washed with Tris-buffered saline containing 0.1% Tween 20 (TBST) 3 times, incubated with the second antibodies (conjugated with peroxidase) for 1 h at room temperature, washed with TBST 3 times. The immunoblots on the membrane were developed with the enhanced chemiluminescence and photographed with an imaging station.

Preparation of protein extracts

Cells were collected from relevant experiments. The cells were incubated with a lysing buffer for 30 min. The lysates were centrifuged for 10 min at 13,000 rpm. The supernatant was collected and used as the cytosolic extracts. The pellets were incubated with a nuclear lysing buffer for 30 min. The lysates were centrifuged for 10 min at 13,000 rpm. The supernatant was collected and used as the nuclear extracts. All the procedures were performed at 4 °C.

Co-immunoprecipitation (co-IP)

The protein samples were precleared by incubating with protein G agarose beads for 30 min to remove pre-existing immune complexes. After centrifugation for 3 min at 13,000 rpm, the supernatant was collected and incubated with antibodies of interest or isotype IgG overnight. The immune complexes in the samples were precipitated by incubating with protein G agarose beads for 30 min with mild agitation. The beads

were collected by centrifugation of the samples at 13,000 rpm for 3 min. The immune complexes on the beads were eluted with an eluting buffer. The proteins were analyzed by Western blotting. All the procedures were performed at 4 °C.

Chromatin IP (ChIP)

Cells were collected from relevant experiments and fixed with 1% formalin for 15 min to cross-link DNA and surrounding proteins. The cells were lysed with a lysing buffer containing protease inhibitor. The DNA in the samples was sheared to small pieces by sonication. The samples were centrifuged at 13,000 rpm for 10 min. The supernatant was collected and processed with the procedures of IP. DNA was extracted from the protein/DNA complexes using a DNA extracting kit following the manufacturer's instructions. The DNA was analyzed by qPCR in the presence of a pair of FasL promoter primers (aggcagagtggtcggtttta and cctatccatcccacttcccc). The results were normalized as fold change against the input. All the procedures were performed at 4 °C.

Assessment of intestinal epithelial barrier permeability in mice

Sixteen hours after treating with C48/80 (ip), mice were treated with oral gavage with 200 µl of TRITC-dextran (40 mg/ml TRITC-dextran 40 kDa in PBS). The mice were sacrificed 4 h post-gavage; blood samples were collected. Levels of circulating TRITC signal were measured by fluorometry.

Flow cytometry

For the surface staining, cells were stained with fluorescence-labeled antibodies of interest or isotype IgG at 4 °C for 30 min. In the case of intracellular staining, cells were fixed with 1% paraformaldehyde (containing 0.1% Triton X-100 to increase the cell membrane permeability) for 1 h. The cells were then stained with fluorescence-labeled antibodies of interest or isotype IgG at 4 °C for 30 min. The cells were analyzed with a flow cytometer (FACSCanto II). The data were processed with the software flowjo with the data obtained from isotype IgG staining as gating references.

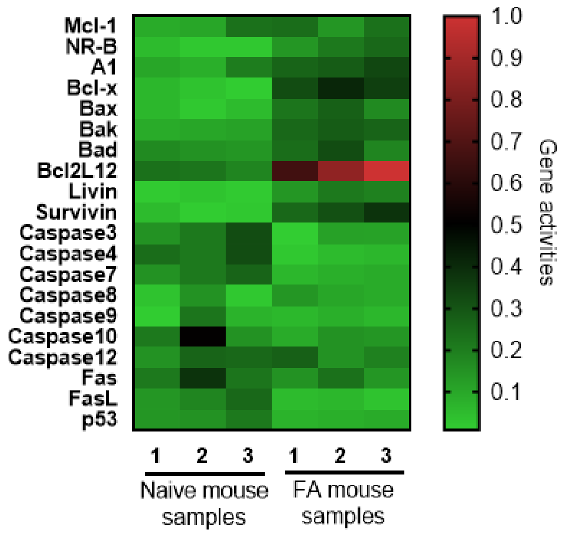

Figure S1. Assessment of apoptosis gene activities in mast cells. Intestinal mast cells were isolated from naïve mice and food allergy (FA) mice by flow cytometry cell sorting. The transcriptome analysis of apoptosis-related genes in the mast cells was carried out by the Ke En Biotech (Shenzhen, China). The heat map shows the

apoptosis-related gene activities in intestinal mast cells of naïve mice (n=3) and FA mice (n=3).

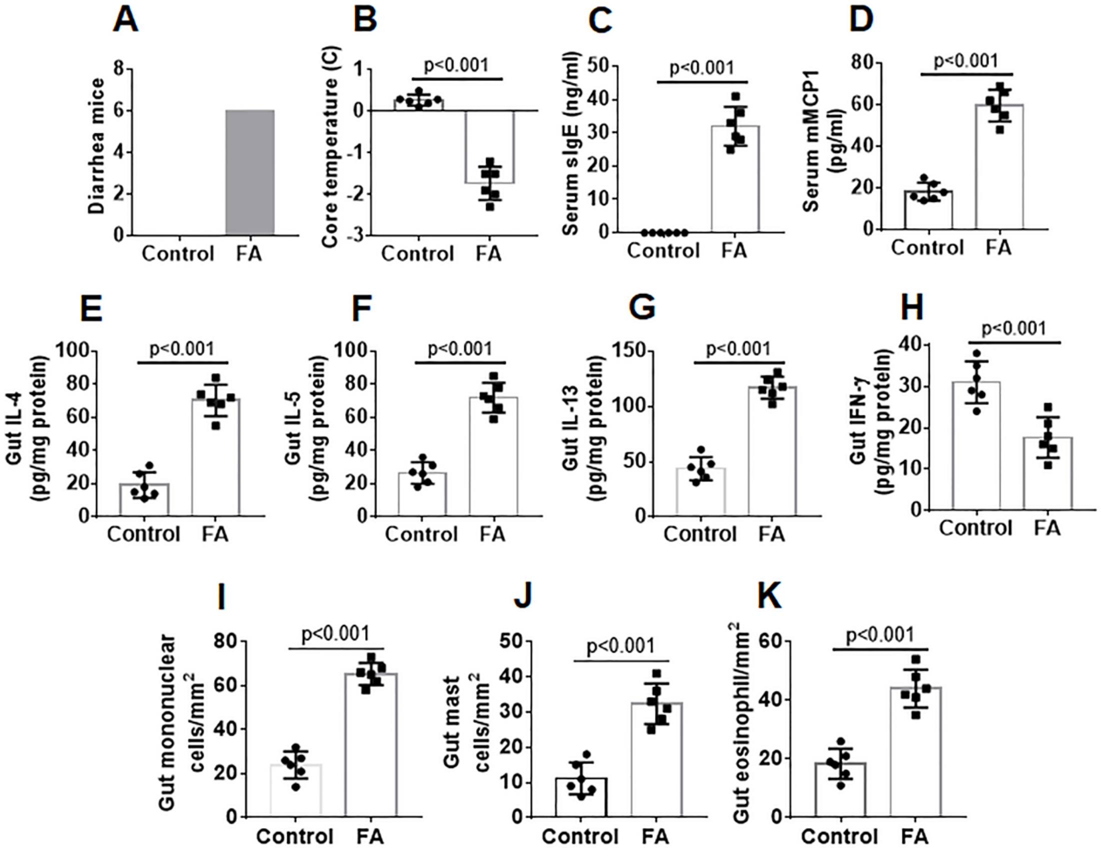

Figure S2. Assessment of allergic status in the intestine of mice. Mice were treated with saline (control group) or OVA (1 mg/mouse)/CT (20 μg/mouse) by gavage-feeding once a week for 4 consecutive weeks. The mice were challenged with 5 mg OVA by gavage in week 5 and sacrificed next day. A, number of mice that had diarrhea after antigen challenge. B, core temperature, recorded 30 min after antigen challenge. C-D, bars indicate serum levels of OVA-specific IgE (sIgE; C) and mouse mast cell protease-1 (mMCP-1; D). E-H, bars indicate levels of Th1/Th2 cytokines in protein extracts of small intestinal tissue. I-K,bars indicate frequency of mononuclear

cells (I), mast cells (J) and eosinophils (K) in the intestinal mucosa. Each group consists of 6 mice. Data of bars are presented as mean ± SEM. Each dot represent data from one mouse.

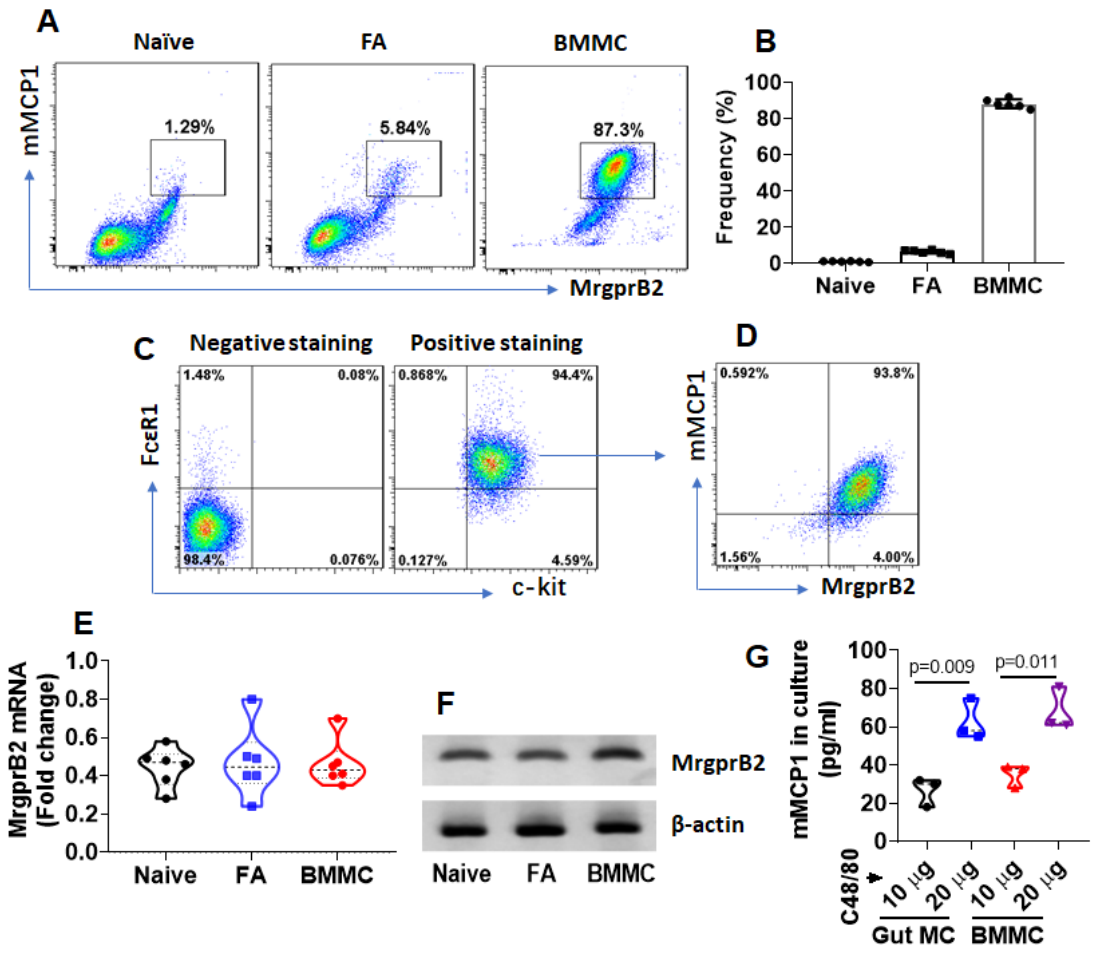

Figure S3. Mouse intestinal mast cells express MrgprB2. Lamina propria mononuclear cells (LPMCs) were isolated from the intestine of naïve mice (n=6) and FA mice (n=6). Bone marrow-derived mast cells (BMMCs) were prepared. A, flow cytometry analysis show mouse mast cell protease-1 (mMCP1)$^{+}$ mast c

ells express MrgprB2 (the gated cells) in LPMCs and BMMCs. B, summarized frequency of MrgprB2^{+} mast cells in LPMCs or BMMCs of panel A. C, FcεRI^{+} c-Kit^{+}

mast cells were isolated from LPMCs by flow cytometry cell sorting. The isolated mast cells were also MrgprB2$^+$ mMCP1$^+$ (D). E-F, the cells were analyzed by RT-qPCR and Western blotting. E, MrgprB2 mRNA levels in isolated mast cells and BMMCs. F, MrgprB2 protein levels. G, levels of mMCP1 in culture supernatant, of which isolated mast cells and BMMCs were stimulated with C48/80 in culture at indicated doses. Data of bars of panel, B, E and G are presented as mean ± SEM. Each dot presents data obtained from one mouse (assessed in duplicate) or one independent experiment (BMMCs).

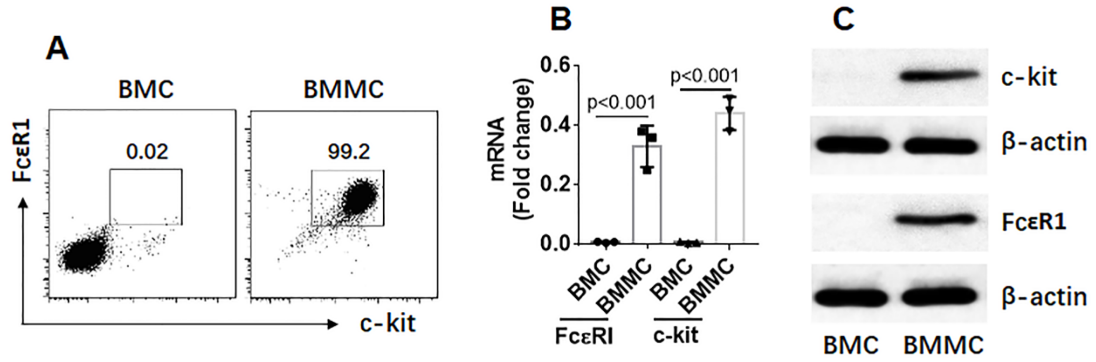

Figure S4. Generation of BMMCs. BMMCs were prepared as described in methods. A, gated dot plots indicate c-kit$^+$ FcεRI$^+$ BMMCs (by flow cytometry). B, bars indicate mRNA levels of FcεRI and c-kit in BMMCs (by RT-qPCR). C, immunoblots indicate protein levels of FcεRI and c-kit in BMMCs (by Western blotting). Data of A and C were from one experiment representing 3 independent experiments. Bars of panel B are presented as mean ± SEM. Each dot represents data from one experiment.

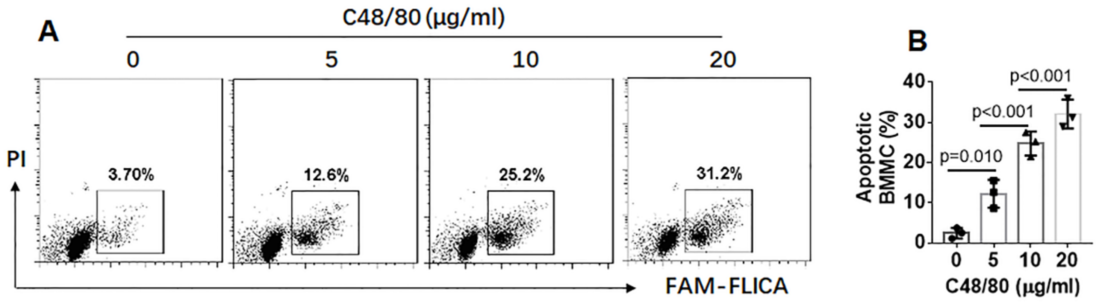

Figure S5. C48/80 induces BMMCs apoptosis. BMMCs were treated with C48/80 at indicated concentrations in the culture for 24 h and analyzed by flow cytometry. A, gated dot plots indicate frequency of apoptotic BMMCs. B, bars (mean ± SEM) indicate summarized data of apoptotic BMMCs of panel A. Each dot represents data from one experiment.

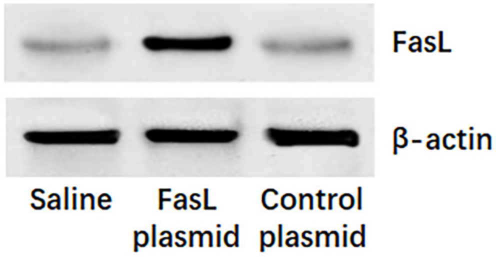

Figure S6. Over expression of FasL in BMMCs. BMMCs were prepared. FasL-expression plasmids and control plasmids were provided by Sangon Biotech (Shanghai, China). The plasmids were transfected into BMMCs following the manufacturer's instructions. Effects of the transfection were assessed by Western blotting 48 h after. The immunoblots indicate FasL levels in the BMMCs. Data are from one experiment that represent 3 independent experiments.

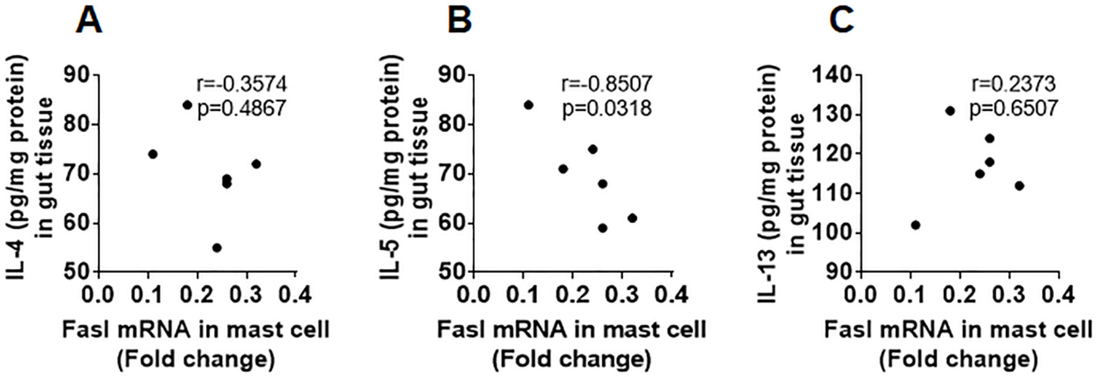

Figure S7. IL-5 negatively correlates with FasL expression in mouse intestinal mast cells. Scatter dot plots show correlation between FasL mRNA in intestinal mast cells (data are presented in Figure 2) and protein of Th2 cytokines (presented in Figure S1E-G). Each dot represent data from one mouse.

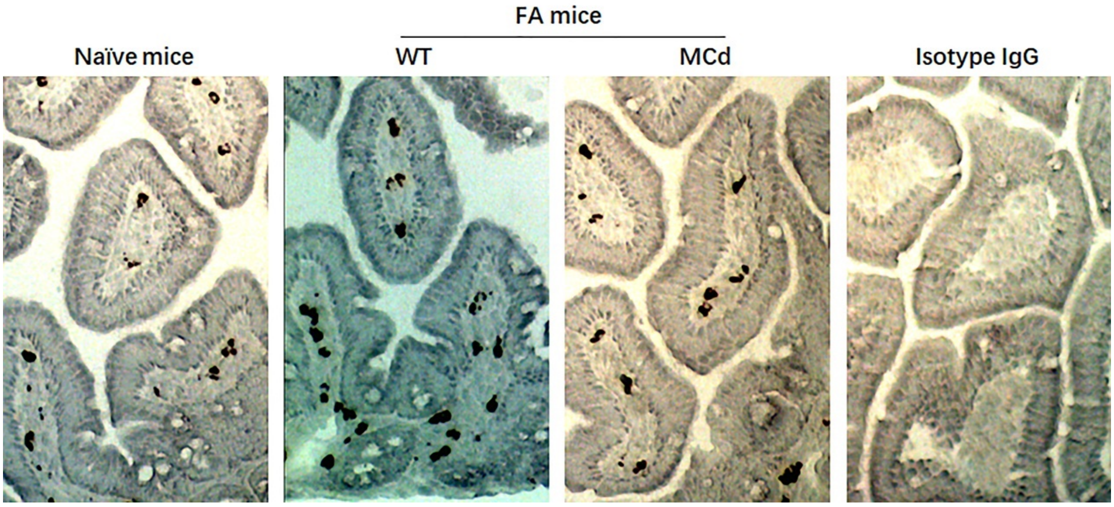

Figure S8. Mast cell counts in the mouse intestine. FA mice were stimulated with C48/80 and re-stimulated with C48/80 two days later. Mast cells were stained in cryosections of small intestine by immunohistochemistry. Representative micro-images show positively stained mast cells (in dark brown) in the intestine. Mast cells were counted in 20 randomly selected microscope windows per sample.

The summarized counts of mast cells are presented in Fig 6E. Original magnification: ×400.

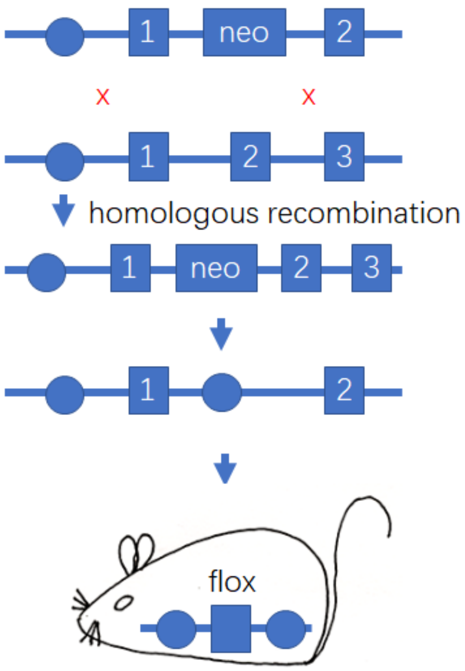

Figure S9. Generation of mouse model of mast cell specific BcL2L12 deletion

To avoid affect the role of Bcl2L12 in other cells, Bcl2L12 was specifically deleted from mast cells with c-kit as the specific marker. Following published procedures (Elife. 2014;3:e01949 and J Clin Invest. 1996;98(3):600-3), we generated mice with loxP-flanked Bcl2L12 gene (flox). In the first step, we constructed a gene targeting vector containing three loxP sites, in which two of them flanking the neomycin resistance gene. The genomic locus was modified between vector and Bcl2L12 gene in embryo stem (ES) cells by homologous recombination. The loxP-flanked neo gene was deleted by transient c-kit-Cre expression in ES cells with two loxP sites remained in target gene. Using the modified ES cells, a loxP-Bcl2L12 containing mouse line was generated. Then, we crossed the mouse strain harboring two loxP sites in the Bcl2L12

gene with another strain expressing c-kit-Cre recombinase. Thus, only in cells expressing c-kit-Cre the Bcl2L12 gene becomes inactivated but remained active in other cells of the body.
